# Structural Characterization of an *S*-enantioselective Imine Reductase from *Mycobacterium Smegmatis*

**DOI:** 10.3390/biom10081130

**Published:** 2020-07-31

**Authors:** Timo Meyer, Nadine Zumbrägel, Christina Geerds, Harald Gröger, Hartmut H. Niemann

**Affiliations:** 1 Structural Biochemistry, Faculty of Chemistry, Bielefeld University, Universitätsstraße 25, 33615 Bielefeld, Germany; timo.meyer@uni-bielefeld.de (T.M.); christina.geerds@uni-bielefeld.de (C.G.); 2 Industrial Organic Chemistry and Biotechnology, Faculty of Chemistry, Bielefeld University, Universitätsstraße 25, 33615 Bielefeld, Germany; nadine.zumbraegel@bayer.com (N.Z.); harald.groeger@uni-bielefeld.de (H.G.)

**Keywords:** biocatalysis, crystal structure, enzyme, 2-ethylhexanol, imine reductase, substrate binding, substrate soaking

## Abstract

NADPH-dependent imine reductases (IREDs) are enzymes capable of enantioselectively reducing imines to chiral secondary amines, which represent important building blocks in the chemical and pharmaceutical industry. Since their discovery in 2011, many previously unknown IREDs have been identified, biochemically and structurally characterized and categorized into families. However, the catalytic mechanism and guiding principles for substrate specificity and stereoselectivity remain disputed. Herein, we describe the crystal structure of *S*-IRED-*Ms* from *Mycobacterium smegmatis* together with its cofactor NADPH. *S*-IRED-*Ms* belongs to the *S*-enantioselective superfamily 3 (SFam3) and is the first IRED from SFam3 to be structurally described. The data presented provide further evidence for the overall high degree of structural conservation between different IREDs of various superfamilies. We discuss the role of Asp170 in catalysis and the importance of hydrophobic amino acids in the active site for stereospecificity. Moreover, a separate entrance to the active site, potentially functioning according to a gatekeeping mechanism regulating access and, therefore, substrate specificity is described.

## 1. Introduction

Ever since the U.S. Food and Drug Administration (FDA) issued regulatory guidelines for the development of stereoisomeric drugs in 1992 [1], the search for a cost-effective (and in recent history also preferably ecologically sustainable) way to generate enantiomerically pure compounds has become a primary concern for the pharmaceutical industry. Beginning with (*S*)-(+)-Ibuprofen in 1994, many chirally switched or de novo synthesized enantiomerically pure drugs have been introduced into the market, leading to 44% of FDA-approved small-molecule active pharmaceutical ingredients (APIs) in 2015 containing one or more chiral centers [2].

Chiral amines are ubiquitously present in nature and among them, secondary amines play an important role. For example, heterocyclic, sulfur-containing amines, in particular 3-thiazolidines, are used in a broad spectrum of pharmaceuticals, most notably as intermediates in the synthesis of β-lactam antibiotics, but also in compounds with anti-inflammatory, antiviral or anti-cancerous effects [3,4]. Consequentially, the asymmetric synthesis of chiral secondary amines has also become one major focus of the industry and many chemical methods to achieve this goal based on the reduction of the C=N bond exist, for example the asymmetric hydrogenation of prochiral imines with transition metal catalysts or stable organic hydrogen donors [5,6]. These methods often suffer from major drawbacks like high costs due to the need for higher amounts of expensive catalysts and hazardous reaction conditions. Furthermore, 3-thiazolidines proved to be all but inaccessible by established reduction technologies as of yet, those failing because of catalyst-poisoning, low reactivity, undesired ring-opening of the product or low enantioselectivity and yields [7,8].

In search for reliable and more environmentally friendly methods applicable under milder conditions, biocatalytic approaches have been increasingly pursued after the discovery of enzymes capable of catalyzing the asymmetric reduction of C=N bonds, most prominently the *Candida albicans* dihydrofolate reductase [9]. In the early 2010s, Mitsukura et al. discovered, purified and characterized NADPH-dependent imine reductases (IREDs) from *Streptomyces* strains, a new enzyme class capable of the conversion of heterocyclic prochiral imines to chiral amines with high enantioselectivity [10,11]. Based on their results, extensive data mining based on sequence similarity revealed hundreds of new, previously unknown IREDs [12]. Crystallographic studies so far indicate a close similarity in the overall structure of these enzymes. They consist of an N-terminal Rossmann-fold and a C-terminal helical domain, connected by a long interdomain helix, and constitute a catalytically active dimer by reciprocal domain sharing [13,14,15,16,17,18,19].

In our recent study we examined several IREDs for their capability of reducing sulfur-containing heterocyclic imines, mainly different 3-thiazolines and 2*H*-1,4-benzothiazines. Out of all the IREDs we tested, the *S*-enantioselective IRED from *Mycobacterium smegmatis* (*S*-IRED-*Ms*), a member of the IRED superfamily 3 (SFam3) first characterized by Wetzl et al. in 2015 [20,21], proved to be the only one capable of reducing both 3-thiazolines and 2*H*-1,4-benzothiazines with promising activity and excellent enantioselectivity [22].

With the prospect of using IREDs as a broadly applicable reduction platform for heterocyclic imines in general, substrate promiscuity with retained enantioselectivity is a desirable property, but for further optimization of the system a deeper understanding of the underlying structural factors influencing substrate specificity and enantioselectivity is necessary. In order to further elucidate this, we present a high-resolution crystal structure of *S*-IRED-*Ms* together with its cofactor NADPH, the first published structure of an SFam3 IRED.

## 2. Materials and Methods

### 2.1. Expression of S-IRED-Ms

The imine reductase from *Mycobacterium smegmatis* (*S*-IRED-*Ms*) was expressed in *E. coli* BL21(DE3) according to a literature-known procedure [21,22]. A preculture of *E. coli* BL21(DE3) carrying the recombinant plasmid with the gene coding for *S*-IRED-*Ms* was cultivated overnight at 37 °C in 10 mL lysogeny broth (LB) medium containing 100 µg/mL carbenicillin. The main culture (6 times 300 mL autoinduction (AI) medium containing 100 µg/mL carbenicillin) was inoculated with the preculture to a final concentration of 1% and cultures were incubated for 2 h at 37 °C and 180 rpm, and afterwards at 15 °C and 180 rpm for 60–70 h. The cells were harvested by centrifugation at 4000× *g* and 4 °C for 30 min and then resuspended in 3 mL 50 mM KP_i_ pH 7.0 containing 20 mM imidazole and 300 mM NaCl per gram of wet cell weight. Cell disruption was performed by homogenisation with the homogenizer EmulsiFlex-C5 (Avestin, Ottawa, Canada) at a pressure of 1000–1500 bar in 3 cycles and the crude extract was obtained as supernatant after centrifugation at 20,000× *g* and 4 °C for 30 min.

### 2.2. Protein Purification

Nickel-nitriloacetic acid (Ni-NTA) affinity chromatography was performed as first purification step. The gravity flow column was packed with Ni-NTA (6–7 mL), washed with 25 mL of Milli-Q ^®^ water (Merck, Darmstadt, Germany) and then equilibrated with 25 mL of washing buffer I (50 mM KP_i_ pH 7.0, 300 mM NaCl, 20 mM imidazole). Crude cell extract (up to 20 mL) was loaded onto the column, resuspended with the column material and incubated for 5 min. The column was washed with 25 mL of washing buffer I and then 50 mL of washing buffer II (50 mM KP_i_ pH 7.0, 300 mM NaCl, 40 mM imidazole). The *S*-IRED-*Ms* was eluted with 10 mL of elution buffer (50 mM KP_i_ pH 7.0, 300 mM NaCl, 300 mM imidazole). The elution fraction was concentrated to a volume of 2.5 mL using a Vivaspin ultrafiltration column with a molecular weight cut-off of 20 kDa. Desalting was performed with PD-10 columns (GE Healthcare, Berlin, Germany) equilibrated with 50 mM KP_i_ pH 7.0 according to the manufacturer’s instructions.

Anion-exchange chromatography using the HiLoad 26/10 Q-Sepharose High Performance column and the ÄKTA ™ system (GE Healthcare) was performed with 50 mM KP_i_ pH 7.0 and a salt gradient of 0 to 1 M NaCl over 10 column volumes.

Size exclusion chromatography was performed with 10 mM HEPES pH 7.0 using the Superdex 200 Increase 10/300 GL column and the ÄKTA ™ system (GE Healthcare). The *S*-IRED-*Ms* fraction was concentrated using a Vivaspin ultrafiltration column (Sartorius, Göttingen, Germany) with a molecular weight cut-off of 20 kDa. The concentration of the purified enzyme (ε = 19,480 M^−1^ cm^−1^) was determined spectrophotometrically and the success of the purification over the three steps was checked by SDS-PAGE (Figure S9). The homogeneity of the purified protein was assessed and verified by mass spectrometry, with *m*/*z* calculated for *S*-IRED-*Ms* to be [M]^+^: 30,996.81 and found to be 30,996.94.

### 2.3. Protein Crystallization

Apo-*S*-IRED-*Ms* as well as holo-*S*-IRED-*Ms* with 5 mM of either NADP^+^ or NADPH were mixed with 1 mM EDTA and 1 mM iodoacetamide for protease inhibition, centrifuged for 30 min at 31,500× *g* and then subjected to broad crystallization trials using a range of commercially available screens.

No crystals were obtained for apo-*S*-IRED-*Ms*. Initial holo-*S*-IRED-*Ms* crystals grew with NADPH at a protein concentration of 10 mg/mL in an optimized crystallization condition A8 of the JCSG+ suite by QIAGEN (0.2 M sodium formate, 20% (*w*/*v*) PEG 3350) in a sitting drop setup with 200 nL protein and 100 nL reservoir solution at 4 °C. Larger crystals for diffraction experiments were obtained after one week at the same protein concentration and temperature in an optimized sitting drop setup with 2 µL protein and 1 µL reservoir solution consisting of 20% (*w*/*v*) PEG 3350 and 0.2 M sodium formate. Crystals were cryoprotected in modified reservoir solution containing 22 % (*w*/*v*) PEG 3350, 0.2 M sodium formate and 20% glycerol and flash-frozen in liquid nitrogen.

Co-crystallization of holo-*S*-IRED-*Ms* with 5–25 mM of various substrates diluted in DMSO (end-concentration 5%) (Table S1) yielded crystals in a wide variety of conditions at 4 °C in a sitting drop setup with 200 nL protein and 100 nL reservoir solution, both with NADP^+^ as well as with NADPH. While co-crystallization with NADPH tended to produce larger, more regularly shaped crystals faster, NADP^+^-containing crystals were generally of equal diffraction quality.

Crystals for anomalous data collection were grown at a protein concentration of 10 mg/mL with 5 mM NADP^+^ and either 5 mM 3-TZ in crystallization condition F5 of the high molecular weight mix PEG smear screen (as described in [23]) or 5 mM BT in crystallization condition H8 of the JCSG+ suite (QIAGEN, Hilden, Germany) (0.2 M sodium chloride, 0.1 M Bis-Tris pH 5.5, 25 % (*w*/*v*) PEG 3350). Crystals were cryoprotected in reservoir solution containing 10 % (*v*/*v*) PEG and flash-frozen in liquid nitrogen.

### 2.4. Data Collection, Processing and Structure Determination

The native synchrotron MX data for holo-*S*-IRED-*Ms* with NADPH were collected at beamline P13 operated by EMBL Hamburg at the PETRA III storage ring (DESY, Hamburg, Germany) [24]. The data were indexed and integrated with the XDS package and scaled to a cut-off resolution of 1.55 Å with AIMLESS from the CCP4 package [25,26,27]. The structure was solved by molecular replacement with Phaser and improved with Phenix Autobuild [28,29]. First, based on solvent content analysis, five copies of an ensemble of the Rossmann fold containing domain of three homologous IREDs (Protein Data Bank (PDB) IDs: 5G6R [17], 3ZGY [13], 4D3S [15]) were placed. Then, two copies of an ensemble of the α-helical domain of three homologous IREDS (PDB IDs: 5G6R, 3ZGY, 4OQY [14]) could be placed sequentially. The three remaining copies of the α-helical domain were placed manually by superposition onto symmetry mates. After rigid body and restrained refinement with phenix.refine [30], the electron density was defined well enough for rebuilding the model in place with the correct amino acid sequence with Phenix AutoBuild [29], greatly improving the free R-factor by 15 % in the process. Subsequently, five copies of NADPH could be placed in the resulting Fo-Fc difference density with Phenix LigandFit [31]. Several rounds of rebuilding and structure validation were performed in Coot [32], followed by rigid body-, coordinate-, TLS-, occupancy- and individual B-factor-refinements in phenix.refine and Refmac5 [33].

Anomalous diffraction data for holo-*S*-IRED-*Ms* with NADPH and either 3-TZ or BT were collected at an energy of roughly 6 keV (wavelengths of 1.9074 Å to 2.0664 Å) on beamline BL14.2 at the BESSY II electron storage ring operated by the Helmholtz-Zentrum Berlin [34], indexed and integrated with the XDS package and scaled with AIMLESS to a cut-off resolution of 2.14 Å for the 3-TZ dataset and 2.40 Å with for the BT dataset. The structures were solved by molecular replacement with Phaser, using one monomer from the previously solved *S*-IRED-*Ms* but without the cofactor NADPH as the initial search model (PDB ID: 6SMT). Cofactor placement, model completion, validation and refinement were per performed analogously to the native dataset. Data collection and refinement statistics are reported in Table 1. Figures were generated with PyMOL (Schrödinger, New York, NY, USA).

## 3. Results

### 3.1. Overall Structure of S-IRED-Ms and Dimer Formation

**Figure 1:**
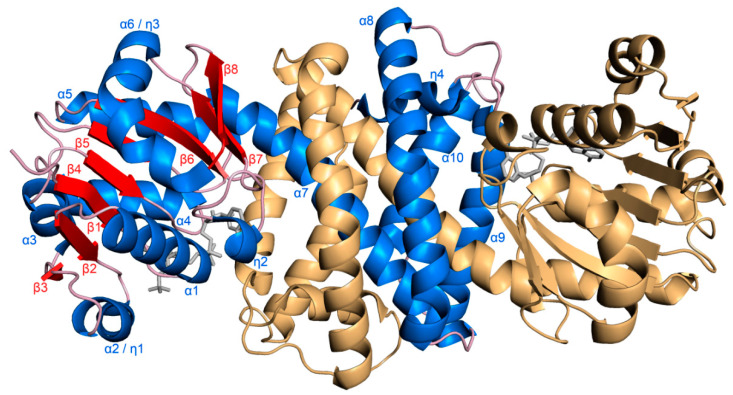
Overall structure of *S*-IRED-*Ms*. *S*-IRED-*Ms,* depicted in cartoon representation, shows the canonical dimeric domain-swapped structure. Helices of monomer A of the dimer are colored and labeled in blue, β-sheets in red, loops and turns in light pink. Monomer B is colored in light orange. The cofactor NADPH is depicted as stick model in gray.

Initially, we tried to crystallize the *S*-IRED-*Ms* (Protein identifier: WP_011731218.1) in the absence of the cofactor NADPH, but these attempts failed to produce diffracting crystals regardless of the presence of substrates. Diffraction-quality crystals were obtained from co-crystallization with NADPH or NADP^+^ alone or with cofactor and one of the various substrates that we had available (Table S1). The *S*-IRED-*Ms* structure presented here (PDB ID: 6SMT) at 1.55 Å resolution was crystallized with NADPH and featured the canonical homodimeric assembly with five monomers (A–E) in the asymmetric unit, arranged as two pairs of dimers (A–B and C–D) and the fifth monomer (E) forming a dimer with its symmetry mate (Figure 1).

The model is of high quality (Table 1) and is mostly complete with the only poor density regions not modeled being the N-terminal methionine, hexahistidine-tag and one (A, B, E) or two (C, D) threonines and the last one (B) to four (A, C-E) amino acids of the C-terminus.

The *S*-IRED-*Ms* monomer, represented by subunit A, consists of an N-terminal Rossmann-fold domain of 160 amino acids (9–169) and a C-terminal helical domain (201–294) connected by a long interdomain helix (170–200). The N-terminal domain consists of a mixed eight-stranded β-sheet interspersed in sequence and encapsulated by six α-helices. The C-terminal domain is comprised of the end of the interconnecting helix and a further four helices (Figure 1 and Figure S1). The dimers are generated by reciprocal domain swapping, where the end of N-terminal domain A, the interdomain helix α7 protrudes and inserts through a hydrophobic channel in the C-terminal domain of chain B (Figure S2) to then emerge and continue as the C-terminal helical domain of A, which contacts the N-terminal domain of subunit B.

Surface-exposed reciprocal salt bridges between R165 (chain A, helix α7) and E200 (chain B, helix α8) at the N-terminal side and between D96 (chain A, loop connecting strand β5 and helix α5) and H244 (chain B, helix α9) as well as a buried salt bridge between D175 (chain A, helix α7) and H186 (chain B, helix α7) at the C-terminal side of the interdomain helix further stabilize the dimer (Figure S3), resulting in a large contact area of 4076 Å² on average per monomer, calculated with the ‘protein interfaces, surfaces and assemblies’ service PISA (http://www.ebi.ac.uk/pdbe/prot_int/pistart.html) [35].

### 3.2. NADPH-Binding Site

The cleft formed between the Rossmann-fold domain of subunit A and the helical domain of subunit B constitutes the active site of *S*-IRED-*Ms* and is a large channel at the dimer interface with two separate openings, presumably for individual binding of NADPH and a substrate, respectively. The electron density obtained after molecular replacement allowed for unequivocal placement of five NADPH molecules in the asymmetric unit, one in each *S*-IRED-*Ms* monomer active site. Albeit not strictly planar, angles in the nicotinamide rings indicate a mixture of NADPH and NADP^+^ in the crystal heavily favoring the reduced form of the cofactor.

**Figure 2:**
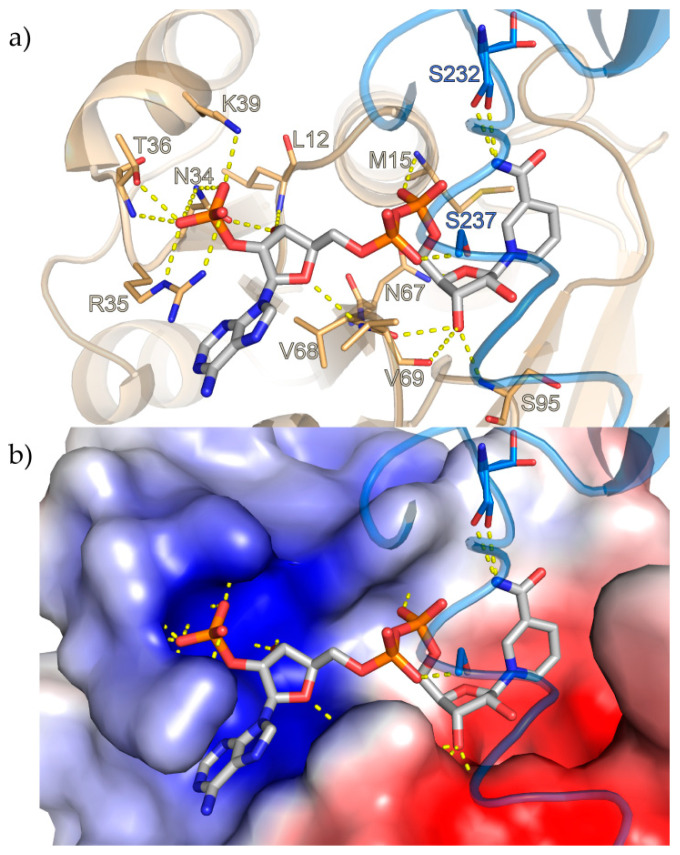
NADPH-binding site of *S*-IRED-*Ms*. NADPH, shown as stick model, resides in a highly conserved binding site. Carbon atoms are depicted in gray, oxygen in red, nitrogen in dark blue and phosphorus in orange. (**a**) Apart from hydrogen bonds to two serines in the C-terminal domain (light blue) NADPH mainly interacts with adjacent residues of the N-terminal Rossmann-fold domain (light orange) and interactions are indicated by dotted yellow lines. (**b**) Visualization of the electrostatic surface potential of the NADPH-binding site displays a positive potential (blue surface) around the adenine moiety of NADPH and its 2′-phosphorylated ribose, while a strongly negative potential (red surface) can be observed in the rest of the channel leading to the substrate binding site. The surface of the C-terminal domain, normally partially covering the NADPH binding site, was omitted from the depiction in order to provide a better view.

The NADPH entrance is situated next to the loop connecting helix α1 to β-strand β1. Apart from hydrogen bonds to S232 and S237, NADPH only interacts with the N-terminal domain. The ribose-2′-phosphate of the adenosine moiety contacts N34, R35, the side-chain and the peptidic NH of T36 and K39, the 3′-ribose hydroxyl interacts with N34 and the peptidic NH of L12 and the ether-oxygen contacts the peptidic NH of V69. The 3′-ribose hydroxyl of the nicotinamide nucleotide interacts with the backbone of S95, V68 and V69, while its 5′-phosphate contacts the peptidic NH of M15 (Figure 2a). The nicotinamide ring is held in place by interactions with S232 and stacks against M15 with its *si*-face presented to the presumed substrate-binding area of the active site. The characteristic GXGXXG consensus sequence for NADPH binding is present as G(11)LGPMG(16) [36].

Calculation of the electrostatic surface potential with the APBS [37] Tool 2.1 plugin in PyMOL revealed that while some of the residues that bind the adenosine moiety of NADPH and its 2′-phosphorylated ribose are positively charged and the cofactor binding site displays a positive electrostatic surface potential, an overwhelmingly negative potential can be observed in the rest of the channel after the diphosphates leading to the substrate binding site and in the site (Figure 2b).

### 3.3. Substrate-Binding Site

**Figure 3:**
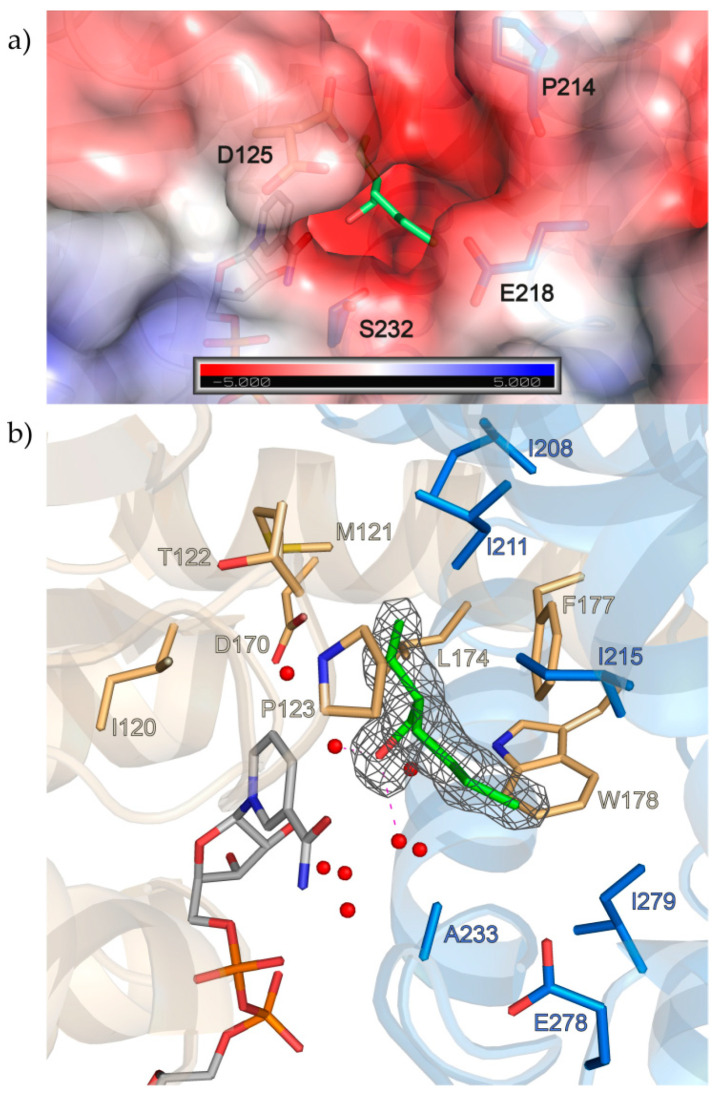
Second entrance and active site of *S*-IRED-*Ms*. (**a**) The opening to the active site cavity shows a negative electrostatic potential. This is largely due to D125, E218 and possibly P214 and S232 outlining the entrance. (**b**) 2-ethylhexanol (2-EH) was modelled into the active site. 2-EH is sandwiched by hydrophobic residues like P123, L174, F177, and W178 and also forms two hydrogen bonds to nearby waters which are part of a wider network inside the active site, further holding 2-EH in place. Amino acids within 5 Å of 2-EH as well as D170 in the proposed protic position are depicted as sticks, with the two chains forming the dimer colored in light orange and marine blue. NADPH is colored in light gray. The 2Fo-Fc electron density of NADPH, contoured at 1 σ, is shown in dark gray. The hydrogen bonds of the 2-EH hydroxyl to nearby water molecules (red spheres) are indicated as pink dashed lines.

The second entrance to the active site in *S*-IRED-*Ms*, situated between the loop connecting the β-strands β6 and β7 of one *S*-IRED-*Ms* monomer and the loop connecting α-helices α8 and α9 as well as helix α8 itself of the other monomer is characterized by a strongly negative electrostatic surface potential, largely due to D125, E218 and possibly to a lesser extent P214 and S232 outlining the entrance (Figure 3a).

Interestingly, diffraction quality crystals could only be obtained with cofactor present in the crystallization cocktail. We observed positive difference electron density inside the active site of the enzyme at a position equivalent to that of substrate molecules in other IRED structures in the literature [16,17,18,19], which was of a similar shape in all the crystal structures we obtained, independent of whether we used NADPH or NADP^+^, which substrate or cryoprotectants we used or if we utilized soaking or co-crystallization methods (Figure S4).

Neither the substrate molecules we screened for, nor any of the other substances contained in the mother liquor could be placed inside this density with confidence. The best fit was achieved with placement of a 2-ethylhexanol (2-EH) molecule, but we do not imply this to be an actual substrate of the enzyme. The origin of this component is unclear, but it is known to be a precursor for the synthesis and also degradation product of the wide-spread plasticizer DEHP [38]. Most known IREDs contain either a tyrosine or an aspartic acid in a conserved position at the top of the site that is demonstrated to play an essential role in catalysis [17,19,20]. The corresponding D170 in *S*-IRED-*Ms* is located 6–7 Å away from the apparent substrate position and this distance is bridged via two water molecules at distances of roughly 2.7 Å and 4.9 Å in between. In addition, subfamily-specific, highly conserved residues presumably mediating stereopreference have been identified in the past [20]. In *S*-IRED-*Ms*, the corresponding residues M121, P123, L174 and F177 are all present within 5 Å of the active site substrate surrogate 2-EH and oriented towards it. Other residues also within 5 Å of the presumed substrate position include I120, T122, W178, I208, I211, I215, A233, GE278 and I279 (Figure 3b).

In some of the datasets we collected from crystals with substrate present in the crystallization cocktails, 2-EH did not seem to fully explain the density observed in the active site. We therefore generated polder maps and composite omit maps to reduce the influence of bulk solvent in phenix, but no substantial improvement of the density could be achieved (Figures S5 and S6) [30,39,40]. We then collected anomalous data of these 2,2,3-trimethyl-1-thia-4-azaspiro[4.4]non-3-ene (3-TZ)- and 2,2,3-trimethyl-2*H*-1,4-benzothiazine (BT)-crystals at an energy of roughly 6 keV to visualize phosphorus and sulfur atoms in order to verify whether the sulfur-containing ligands could be present with partial occupancy or in different orientations due to the size of the active site. While the methionine-sulfurs and NADPH-phosphates were clearly visible, no clear sulfur-signal could be detected in the active site cavity itself, thereby confirming the absence of the sulfur-containing substrates (Supplementary Figure S7).

Other groups in the field previously described the related enzymes *Ao*IRED, *Bc*IRED and *Asp*RedAm switching to a closed conformation upon cofactor binding, characterized by a relative angle-closure between the N-terminal Rossmann- and the C-terminal helical domain and by a loop restricting access to the NADPH-binding site in their respective crystal structures [15,16,17], while others did not observe such movements [13]. Since we did not obtain crystals of apo-*S*-IRED-*Ms*, no direct comparison between the former and the cofactor-bound holo-*S*-IRED-*Ms* can be drawn. The state that holo-*S*-IRED-*Ms* was crystallized in could be best described a closed state, resembling the closed state found in *Asp*RedAm (PDB ID: 5G6S), the reductive aminase most similar to *S*-IRED-*Ms* according to the DALI-Server (http://ekhidna2.biocenter.helsinki.fi/dali) [41], which has a root mean square deviation (r.m.s.d.) to *S*-IRED-*Ms* of 1.3 Å in its closed form and 1.6–1.9 Å in its open form (Figure S8 and Table S2).

## 4. Discussion

In the last ten years, IREDs have become a promising target for the development of a biocatalytic approach to the enantioselective generation of chiral amines via asymmetric reduction of prochiral imines.

IREDs are structurally closely related to the family of β-hydroxy acid dehydrogenases (β-HADs) and with NADPH also being the essential cofactor for both [20], it was assumed they also share a similar reaction mechanism where NADPH acts as a hydride donor while a protic amino acid, in most IREDs either tyrosine or aspartic acid, would act as a proton donor [13].

Hence, two mechanisms have been discussed in the past differing in the sequence of events. In Irp3, a thiazolinyl imine reductase from *Yersinia enterocolitica*, the hydride transfer occurs first, followed by protonation via a general acid residue [42]. However, it has to be taken into account that the C=N double bond of such substrates is embedded into a imido thioester subunit, which means that this C=N double bond is more strongly activated compared to the C=N bond of “typical” imines (Schiff bases) such as the one in the 3-thiazoline considered in this study.

However, the necessity of a proton donor amino acid in IREDs has remained ambiguous to this day. Mutational studies revealed that substitution for a non-protic residue like alanine in this position can in some IREDs lead to retained albeit lower enzymatic activity [12,43]. In addition, a wild-type IRED from *Pseudomonas putida* with alanine in this position has been described [44], suggesting either the existence of additional contributing factors in the protonation process or an altogether different mechanism.

The second proposed mechanism is based on dihydrofolate reductase (DHFR). Here, the substrate is protonated by water and the hydride transfer occurs afterwards, completing the reduction [45]. It has thus been speculated that IREDs do not use this position for proton donation but rather for anchoring and positioning substrate in an optimal orientation and distance to the cofactor NADPH, since they might operate at a pH value where the substrate is already presented as a protonated iminium species [19]. This fits well with our observations, that in most IREDs in general and in *S*-IRED-*Ms* in particular the electrostatic potential of the active site as well as of the second entrance to it is negative, which could therefore act in a gatekeeping manner favoring the entry of positively charged molecules.

Previous findings indicated that several conserved hydrophobic residues on the sides of the active site and not the one in the general acid position are responsible for the enzymes’ enantioselectivity. This is supported by the fact that the *S*-enantioselective *S*-IRED-*Ms*, an SFam3 IRED, shares the aspartic acid in the general acid position with IREDs from SFam1, which are *R*-selective, while the aforementioned conserved hydrophobic residues are shared with SFam2 IREDs, which are also *S*-selective like *S*-IRED-*Ms* but differ in their general acid residue [20].

However, the high substrate promiscuity of these enzymes paired with different substrate specificities of IREDs within the same subfamily remains enigmatic. In our previous study, we tested four other SFam3 IREDs in addition to *S*-IRED-*Ms*, and while sequence alignments reveal that residues around 5 Å of the substrate in the active site seem to be conserved (Figure S1), these IREDs demonstrated next to no activity on the substrates that *S*-IRED-*Ms* converted readily [22], again indicating the presence of a potential gatekeeping mechanism limiting access to the active site.

However, only a few of the hundreds of known IREDs have been structurally described, and it is possible that interactions not yet observed or considered are responsible for IRED reduction characteristics. To fully understand the catalytic mechanism and its underlying principles, it will thus be imperative to obtain more structural data with substrates in place, preferably of other SFam3 IREDs. The apparently complex interactions controlling the processes in the active site, the substrate promiscuity and lack of knowledge with regard to endogenous substrates of IREDs complicate this endeavor. Therefore, it might be necessary to stabilize the transitional state in which the substrate is bound to the enzyme in a protonated state and to inhibit the enzyme’s capability to complete the reduction mechanism in order to limit dissolution of the *S*-IRED-*Ms*-substrate complex and stabilize it for crystallization. This could potentially be achieved by utilizing positively charged iminium ions which have been demonstrated to be valid substrates before and redox-inactive cofactor analogues like NADPH_4_ which has been used to crystallize the closely related *At*RedAm with a synthetic substrate [18,43]. Another, albeit more complex strategy to understand the role and mechanism of IREDs could involve identifying their respective endogenous substrates via untargeted and combination metabolomics approaches [46,47].

## Figures and Tables

**Table 1:** Data collection and refinement statistics of holo-S-IRED-Ms crystallized with its cofactor NADPH.

Data Collection Statistics	Holo-*S*-IRED-*Ms*
Beam line	DESY P13
Detector	DECTRIS PILATUS 6M-F
Wavelength (Å)	0.9762
Temperature (K)	100
Space Group	I 1 2 1
Unit cell dimensions	a = 117.0 Å
	b = 77.0 Å
	c = 194.5 Å
	α = γ = 90.000 °
	β = 90.204 °
Resolution (highest resolution shell) (Å)	100.42–1.55 (1.58–1.55)
Completeness (%)	99.1 (96.3)
Multiplicity	6.9 (6.4)
Observations	1,702,995 (76,619)
Unique reflections	248,023 (11,889)
I/σ(I)	13.2 (3.2)
R_merge_ (%)	7.3 (38.4)
R_meas_ (%)	8.0 (41.7)
R_pim_ (%)	3.0 (16.2)
CC ½	99.7 (92.8)
Monomers per asymmetric unit	5
Solvent content (%)	53.0
Wilson B-factor (A²)	16.76
**Refinement Statistics**	
Resolution (highest resolution shell) (Å)	100.42–1.55 (1.59–1.55)
Rwork (%) (highest resolution shell)	13.08 (17.30)
Rfree (%) (highest resolution shell)	15.49 (20.20)
Average B-factor (A²)	21.0
protein	18.6
ligands	20.3
solvent	34.2
Number of non-hydrogen atoms	13,314
protein	10,980
ligands	364
solvent	1970
r.m.s.d. bondlength (from ideal geometry)	0.0193
r.m.s.d. angle (from ideal geometry)	2.2672
Ramachandran favored (%)	99.86
Ramachandran allowed (%)	0.14
Ramachandran outlier (%)	0.00
Rotamer outliers (%)	0.98

## Supplementary Materials

The following are available online at https://www.mdpi.com/2218-273X/10/8/1130/s1; Figure S1, Sequence alignment of *S*-IRED-*Ms* and other SFam3-type IREDs; Figure S2, The interdomain helix; Figure S3, Salt bridges stabilizing *S*-IRED-*Ms*; Figure S4, Similar electron density in the active site of various *S*-IRED-*Ms* crystals; Figure S5, Polder density in the active site of various *S*-IRED-*Ms* crystals; Figure S6, Electron density in the active site of various *S*-IRED-*Ms* crystals after substrate placement; Figure S7, 2Fo-Fc, Fo-Fc and anomalous difference density of an *S*-IRED-*Ms* crystal; Figure S8, Comparison of the open and closed state of *Asp*RedAm to *S*-IRED-*M*s; Figure S9, SDS-PAGE of all purification steps of *S*-IRED-*Ms*; Table S1, List of substrates used for crystallization experiments with *S*-IRED-*Ms* in this study [48,49,50,51]; Table S2, List of the ten chains in the PDB most similar to *S*-IRED-*Ms* according to the DALI-Server; [41].
