# Supplementary material for: Structural Characterization of an S-enantioselective Imine Reductase from Mycobacterium Smegmatis
Source: Biomolecules. 2020 Jul 31;10(8):1130. doi: 10.3390/biom10081130 (PMC7465668; doi:10.3390/biom10081130)
Supplement: Supplementary file 1 [file biomolecules-10-01130-s001.pdf]

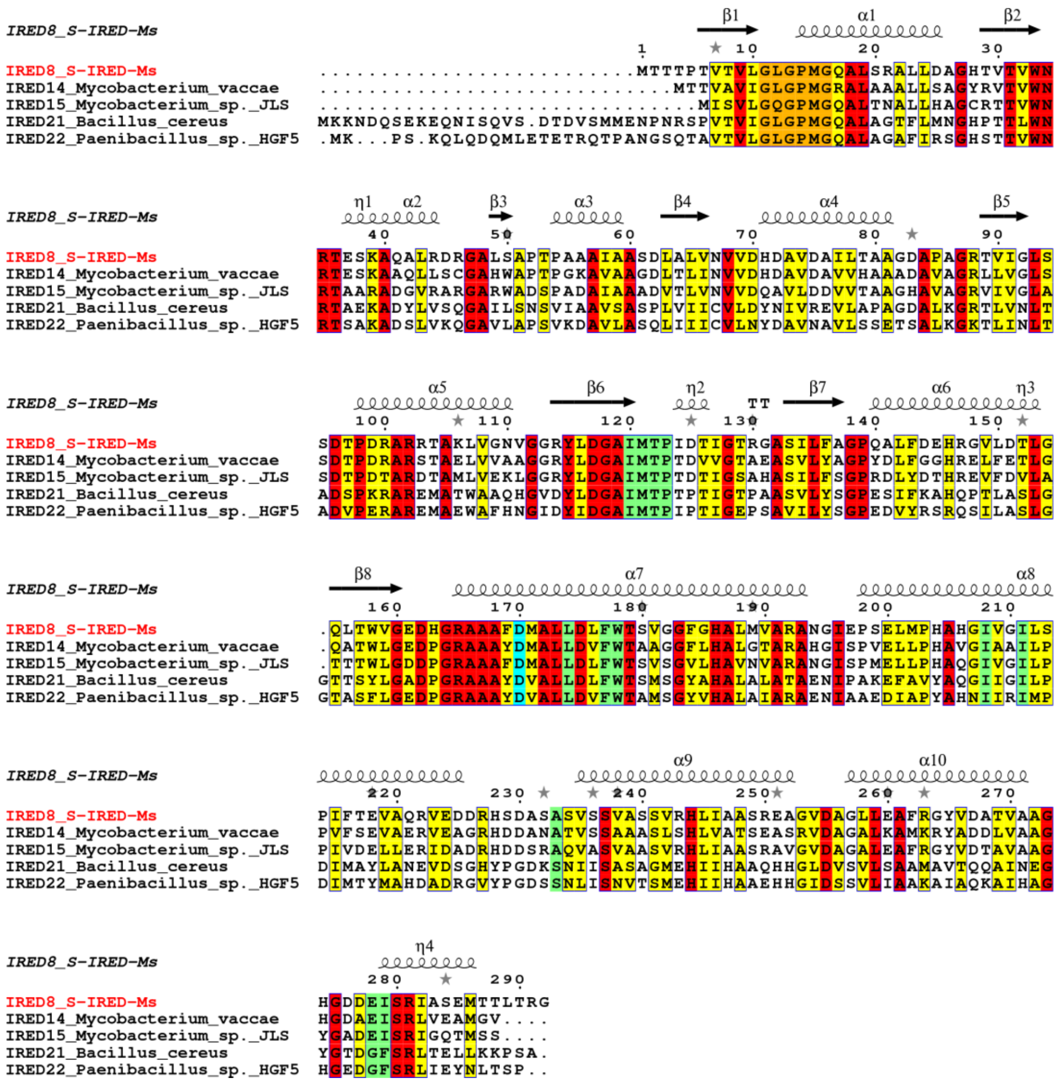

Figure S1: Sequence alignment of *S*-IRED-*Ms* and other SFam3-type IREDs. All IREDs selected for comparison were tested in our previous study and labelling (apart from *S*-IRED-*Ms*) has been done accordingly [22]. Residues of strict identity (red), residues with an overall similarity score greater than 0.7 (yellow), the NADPH-binding motif GxGxxG (orange), residues within 5 Å of the substrate binding position defined by the placement of 2-EH in the electron density (green) and the Asp in the presumed protic position (cyan) are highlighted. Numbering of residues and secondary structure depiction is based on *S*-IRED-*Ms*.

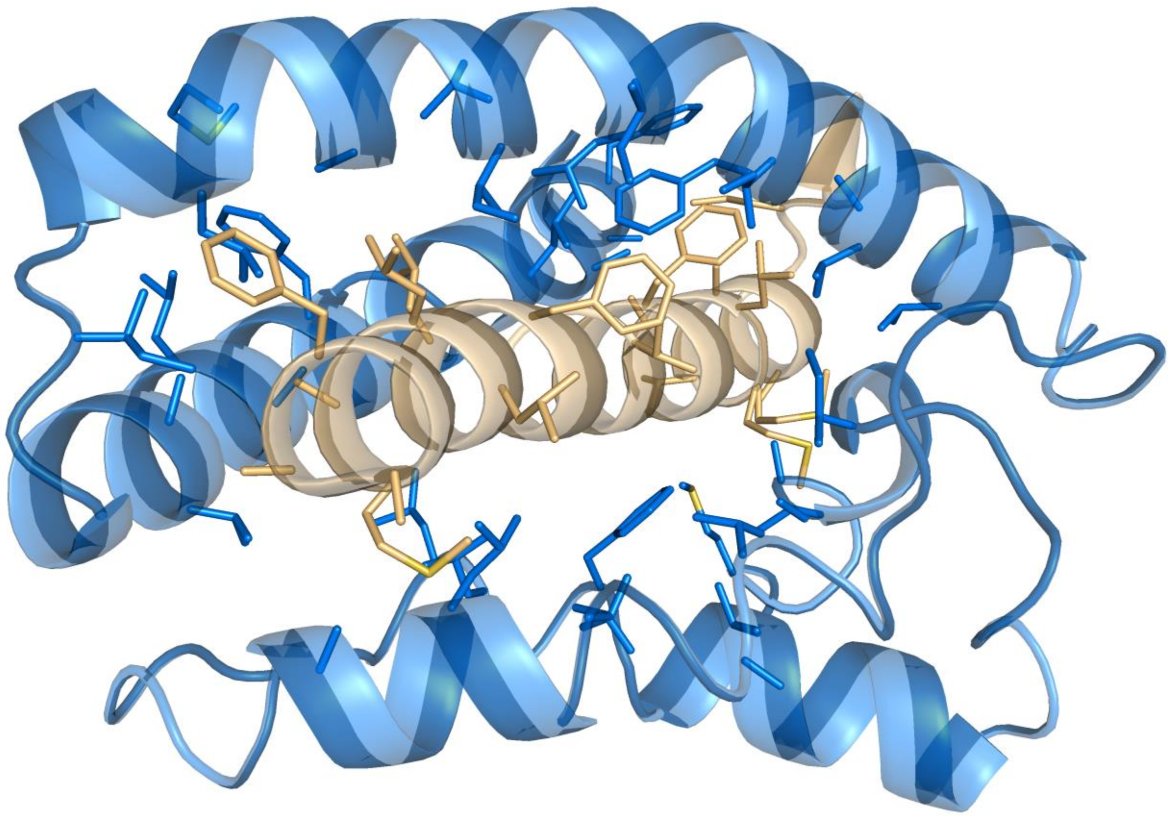

Figure S2: The interdomain helix. The interdomain helix α7 of one monomer passes through a hydrophobic tunnel comprising of helices α7, α8, α9, α10 and η4 of the other monomer.

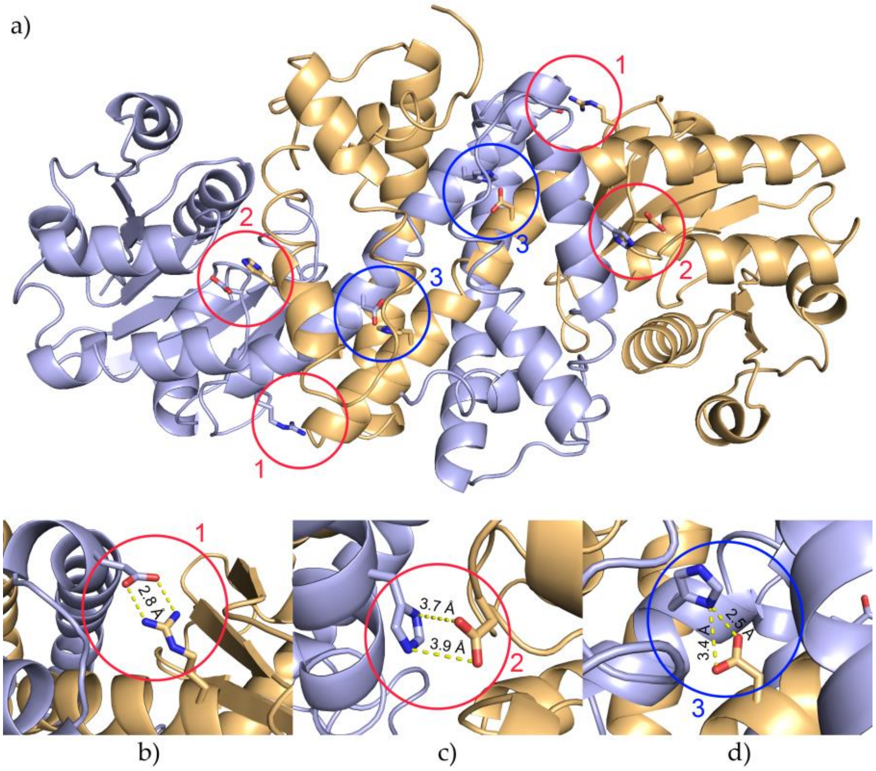

Figure S3: Salt bridges stabilizing *S*-IRED-*Ms*. (**a**) Three salt bridges indicated by the PISA server stabilize the *S*-IRED-*Ms* dimer (monomers in cartoon depiction, light blue and light orange), out of which two are surface-exposed (red, 1 and 2) and one is buried (blue, 3). (**b**), (**c**) and (**d**) provide close-ups of the respective salt bridges.

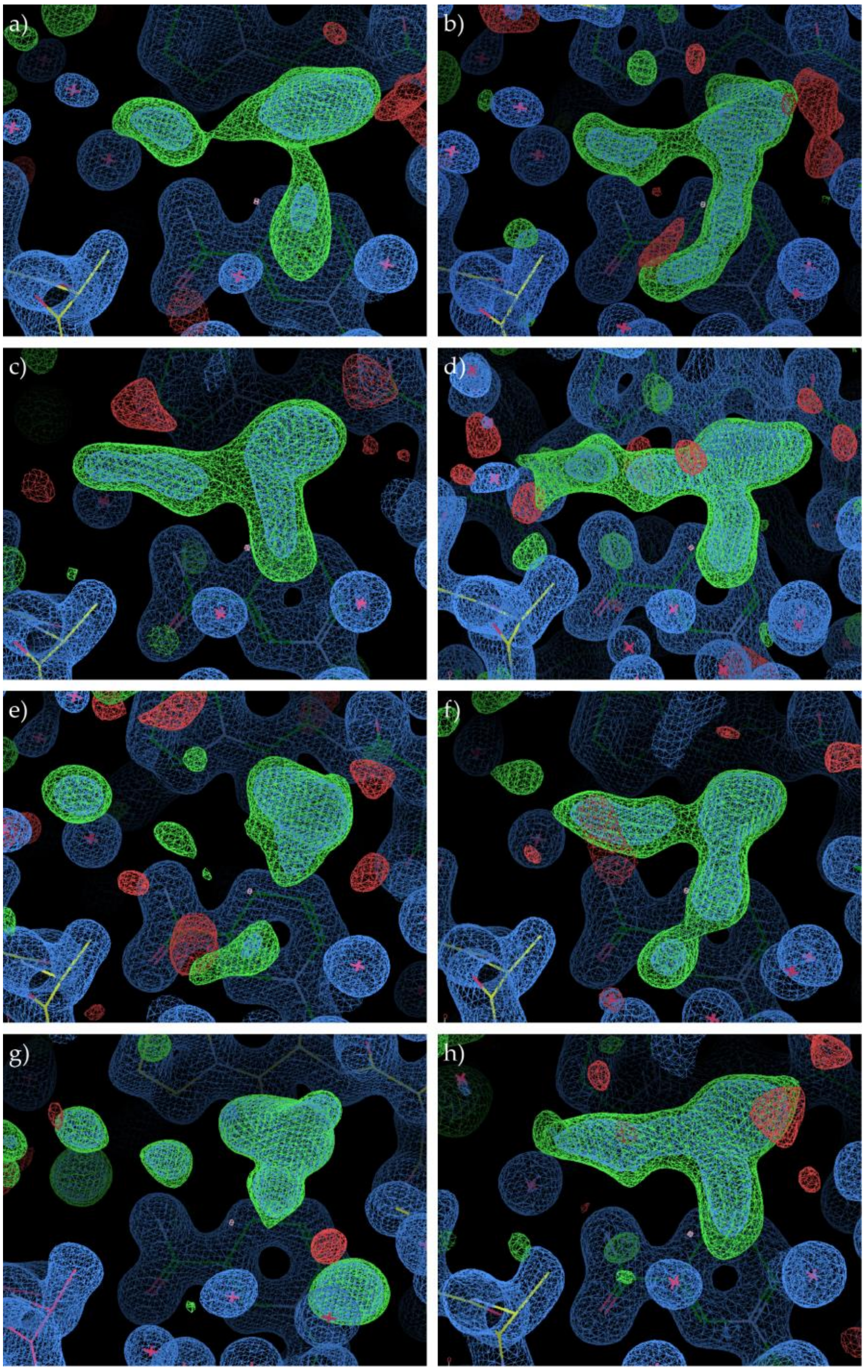

Figure S4: Similar electron density in the active site of various *S*-IRED-*Ms* crystals. All datasets had the protein and NADPH placed and restrained refinements performed on them. The 2Fo-Fc density is contoured in blue at 1.5 σ, the Fo-Fc density in green at +3 σ. The density in the active site shows a similar shape with a strong blob in the upper right and two stalks of varying intensity going down and to the left. *S*-IRED-*Ms* was (**a**) co-crystallized with 5 mM of BO and NADPH, (**b**) co-crystallized with 5 mM of 3-TZ and NADPH, (**c**) co-crystallized with 5 mM of 3-TZ and $NADP^+$, (**d**) co-crystallized with 25 mM of IC and 5 mM of $NADP^+$, (**e**) co-crystallized with 5 mM of BT and NADPH, (**f**) co-crystallized with 5 mM of BT and $NADP^+$, (**g**) crystallized with 5 mM of NADPH and soaked with 5 mM of BT and (**h**) co-crystallized with 25 mM of MP and 5 mM of $NADP^+$.

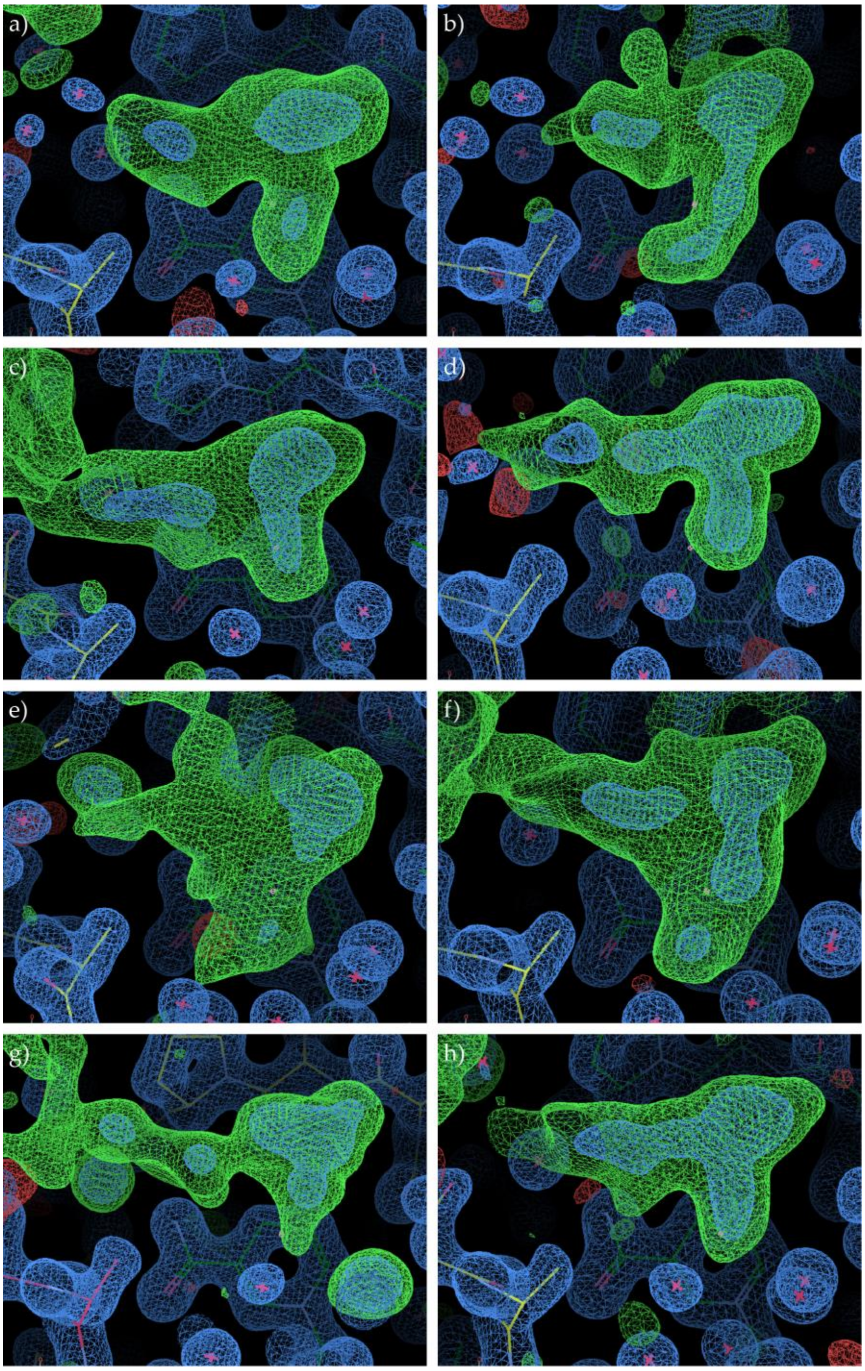

Figure S5: Polder density in the active site of various *S*-IRED-*Ms* crystals. All datasets had the protein and NADPH placed and restrained refinements performed on them. The 2Fo-Fc density is contoured in blue at 1.5 σ, the polder density in green at +3 σ. The polder densities calculated to remove the bulk solvent influence still show a similar shape with a strong blob in the upper right and two stalks of varying intensity going down and to the left, albeit with some more branching. *S*-IRED-*Ms* was (**a**) co-crystallized with 5 mM of BO and NADPH, (**b**) co-crystallized with 5 mM of 3-TZ and NADPH, (**c**) co-crystallized with 5 mM of 3-TZ and $NADP^+$, (**d**) co-crystallized with 25 mM of IC and 5 mM of $NADP^+$, (**e**) co-crystallized with 5 mM of BT and NADPH, (**f**) co-crystallized with 5 mM of BT and $NADP^+$, (**g**) crystallized with 5 mM of NADPH and soaked with 5 mM of BT and (**h**) co-crystallized with 25 mM of MP and 5 mM of $NADP^+$.

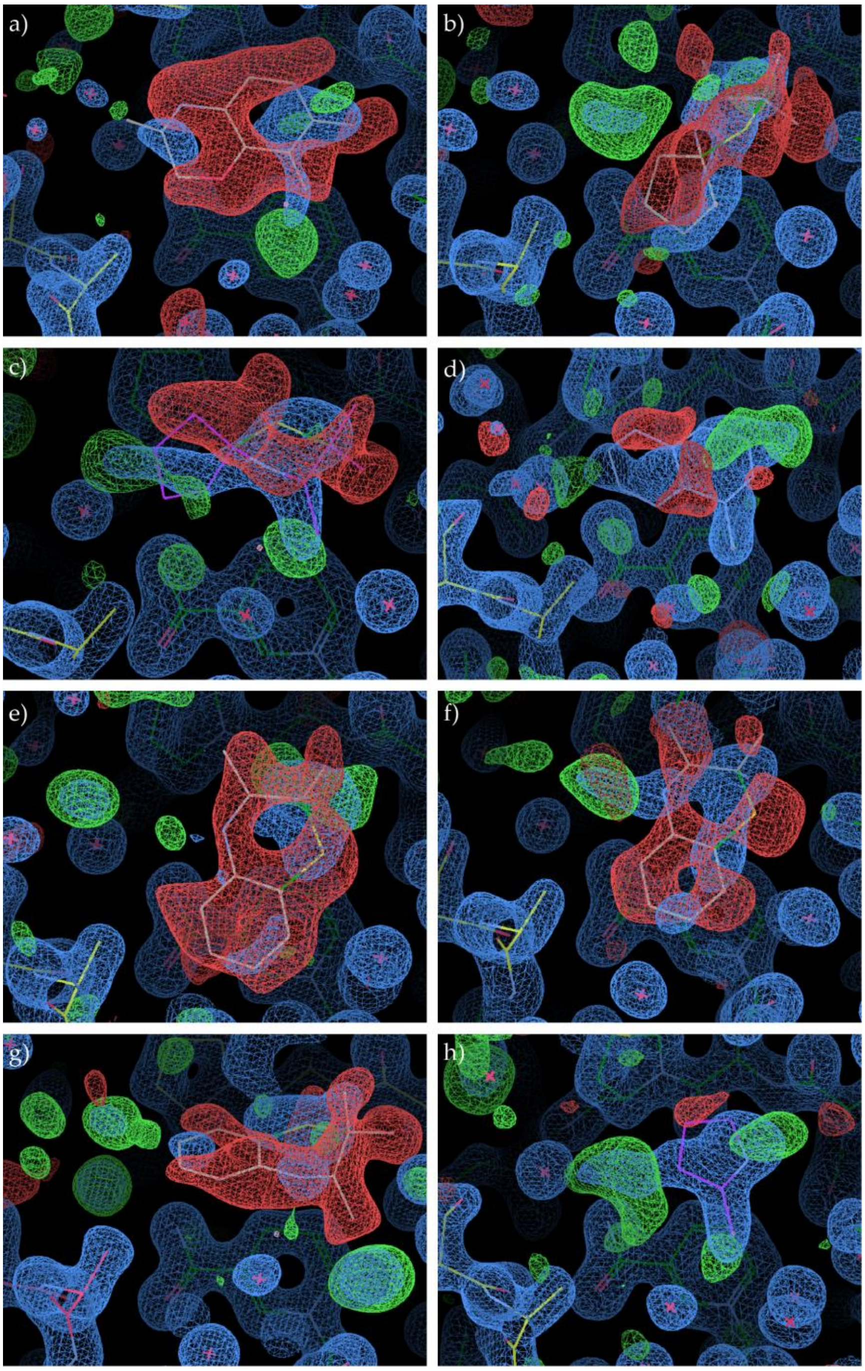

Figure S6: Electron density in the active site of various *S*-IRED-*Ms* crystals after substrate placement. All datasets had the protein and NADPH placed and restrained refinements performed on them, then the polder density of the active site was calculated. Substrates were placed in the polder density to a visually best fit and refined again. The 2Fo-Fc density is contoured in blue at 1.5 σ, the positive Fo-Fc density in green and the negative one in red at 3 σ. The difference density clearly shows, that the substrate molecules cannot explain the electron density observed in the active site. *S*-IRED-*Ms* was (**a**) co-crystallized with 5 mM of BO and NADPH, (**b**) co-crystallized with 5 mM of 3-TZ and NADPH, (**c**) co-crystallized with 5 mM of 3-TZ and $NADP^+$, (**d**) co-crystallized with 25 mM of IC and 5 mM of $NADP^+$, (**e**) co-crystallized with 5 mM of BT and NADPH, (**f**) co-crystallized with 5 mM of BT and $NADP^+$, (**g**) crystallized with 5 mM of NADPH and soaked with 5 mM of BT and (**h**) co-crystallized with 25 mM of MP and 5 mM of $NADP^+$.

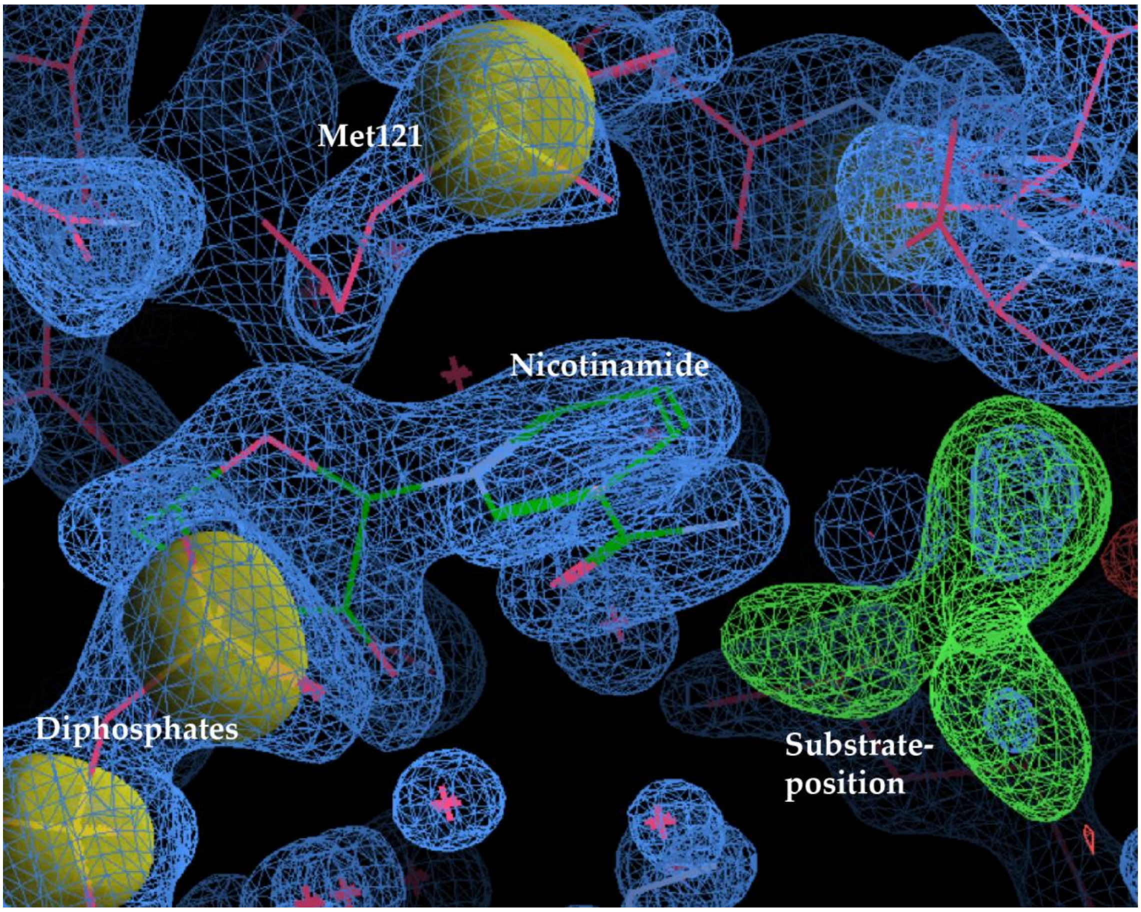

Figure S7: 2Fo-Fc, Fo-Fc and anomalous difference density of an *S*-IRED-*Ms* crystal. The protein was co-crystallized with substrate 3-TZ. The 2Fo-Fc electron is contoured at 2 σ and shown in blue, the Fo-Fc-Density is contoured at +3 σ and shown in green. The difference electron density calculated from the anomalous signal collected at 6 keV is depicted as solid yellow blobs, contoured at +4 σ. While the Met121-sulfur and NADPH-diphosphates can be clearly seen, no anomalous difference density is visible in the substrate-position.

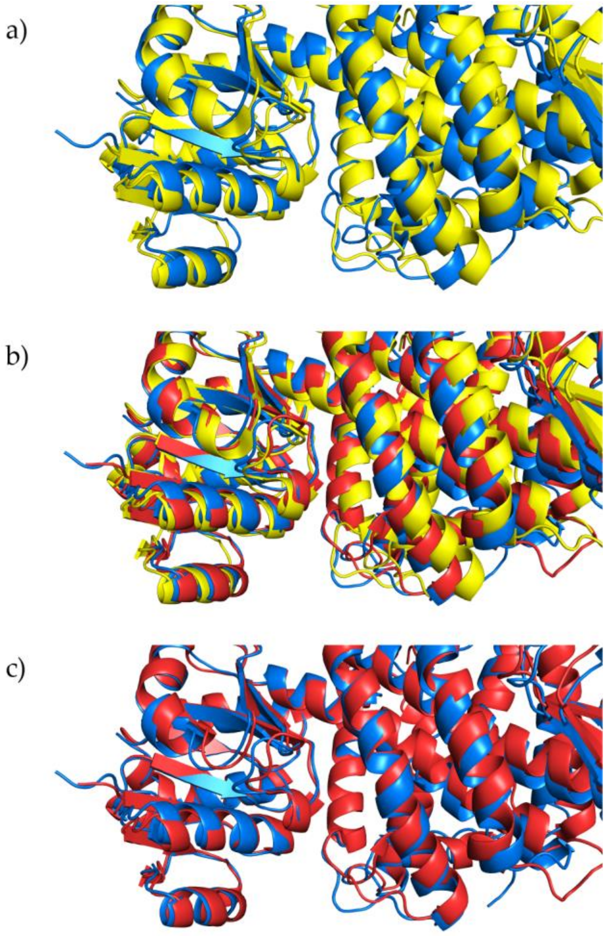

Figure S8: Comparison of the open and closed state of *Asp*RedAm to *S*-IRED-*Ms*. The active site macrostructure of holo-*S*-IRED-*Ms* (blue) in superposition with that of (**a**) *Asp*RedAm in its open form (yellow, PDB ID: 5G6R), that of (**b**) the closed (red, PDB ID: 5G6S) and open form, and (**c**) with *Asp*RedAm in its open form alone. Holo-*S*-IRED-*Ms* more closely resembles the closed state of *Asp*RedAm, also reflected in lower rmsd values of 1.3 Å compared to 1.6-1.9 Å for the open form (see Table S2).

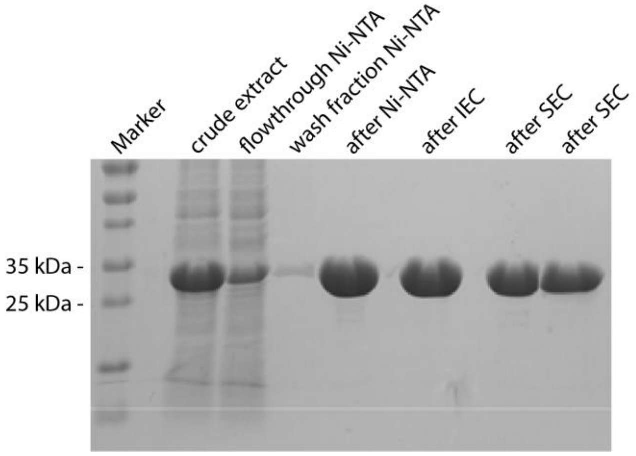

Figure S9: SDS-PAGE of all purification steps of *S*-IRED-*Ms*. 10 μg or 10 μL of sample from the crude extract, Ni-NTA affinity chromatography, anion-exchange chromatography, and size exclusion chromatography were analyzed via SDS-PAGE. Pure protein is visible at 31 kDa.

Table S1. List of substrates used for crystallization experiments with *S*-IRED-*Ms* in this study.

Substrate	Abbreviation	Structure	Source	Soak +NADPH	Soak $+NADP^+$	Co-crystallization +NADPH	Co-crystallization $+NADP^+$
2,2,3-Trimethyl-1-thia-4-azaspiro[4.4]non-3-ene	3-TZ	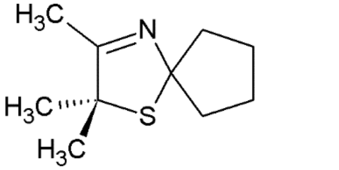	Synthesis as described in:<br><br>Martens, 1981 [48]<br>Reiners, 1997 [7]<br>Zumbrägel, 2018 [22]	-	-	Max. concentration: 5mM<br>Ligand in cryo: y<br>Max. resolution: 1.40<br># data sets: 24	Max. concentration: 5mM<br>Ligand in cryo: y<br>Max. resolution: 1.65<br># data sets: 2
7,8-difluoro-3-methyl-2*H*-benzoxazine	BO	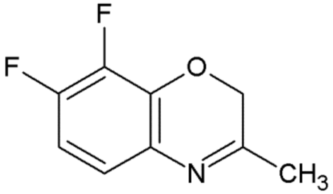	Synthesis as described in:<br><br>Battistoni, 1979 [49]<br>Zumbrägel, 2019 [50]	-	-	Max. concentration: 5mM<br>Ligand in cryo: y<br>Max. resolution: 1.42<br># data sets: 40	Max. concentration: 5mM<br>Ligand in cryo: y<br>Max. resolution: >5<br># data sets: 1
2,2,3-Trimethyl-2*H*-1,4-benzothiazine	BT	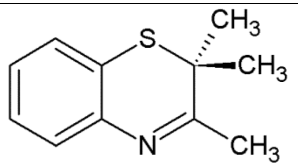	Synthesis as described in:<br><br>Stalling, 2013 [51]<br>Zumbrägel, 2018 [22]	Max. concentration: 5mM<br>Ligand in cryo: y<br>Max. resolution: 1.40<br># data sets: 17	-	Max. concentration: 5mM<br>Ligand in cryo: y<br>Max. resolution: 1.57<br># data sets: 20	Max. concentration: 5mM<br>Ligand in cryo: y<br>Max. resolution: 1.70<br># data sets: 8
1-methyl-3,4-dihydroisoquinoline	IC	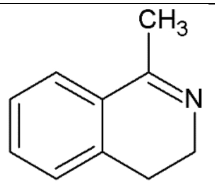	CAS: 2412-58-0 commercially available, used as received	-	-	-	Max. concentration: 25mM<br>Ligand in cryo: y<br>Max. resolution: 1.50<br># data sets: 7
2-methyl-1-pyrroline	MP	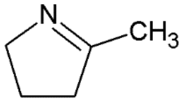	CAS: 872-32-2 commercially available, used as received	-	-	-	Max. concentration: 25mM<br>Ligand in cryo: y<br>Max. resolution: 1.67<br># data sets: 11

Table S2. List of the ten chains in the PDB most similar to *S*-IRED-*Ms* according to the DALI-Server [41].

No	Chain	Z-Score	r.m.s.d.	Lali	nres	%id	PDB description
1	5G6S-G	33.6	1.3	285	289	27	IMINE REDUCTASE
2	5OCM-E	33.4	1.9	283	286	36	NAD_GLY3P_DH, NAD-DEPENDENT GLYCEROL-3-PHOSPHATE
3	6JIT-C	33.0	1.5	286	292	37	6-PHOSPHOGLUCONATE DEHYDROGENASE NAD-BINDING PROT
4	5G6R-A	32.9	1.9	284	288	27	IMINE REDUCTASE
5	6JIZ-C	32.6	1.5	286	295	37	6-PHOSPHOGLUCONATE DEHYDROGENASE NAD-BINDING PROT
6	4D3F-B	32.0	2.3	285	289	32	IMINE REDUCTASE
7	4D3S-F	31.8	1.7	283	283	36	IMINE REDUCTASE
8	4OQZ-A	31.8	1.9	282	283	33	PUTATIVE OXIDOREDUCTASE YFJR
9	3ZHB-A	31.6	2.5	285	288	37	R-IMINE REDUCTASE
10	6EOH-A	31.5	1.5	283	285	34	REDUCTIVE AMINASE
